# Transcriptome analysis reveals an important candidate gene involved in both nodal metastasis and prognosis in lung adenocarcinoma

**DOI:** 10.1186/s13578-019-0356-1

**Published:** 2019-11-19

**Authors:** Xiao Zhu, Hui Luo, Ying Xu

**Affiliations:** 1 Southern Marine Science and Engineering Guangdong Laboratory–Zhanjiang, The Marine Biomedical Research Institute, Guangdong Medical University, Zhanjiang, China; 2 Computational Systems Biology Lab (CSBL), Institute of Bioinformatics, University of Georgia, Athens, GA 30902 USA

**Keywords:** TCGA, Lung adenocarcinoma, Transcriptome, Nodal metastasis, Prognosis, RN7SL494P

## Abstract

Lymph node metastasis of lung cancer is a serious problem. Therefore, there is a need for a detailed transcriptome study of metastatic lung adenocarcinoma. The lung adenocarcinoma RNA-seq data and the corresponding clinical information available from TCGA were analyzed. Differential expression, gradient changes, and biological pathways were carried out. Potential gene(s) associated with tumor metastasis and survival were validated by Cox regression. A total of 406 and 439 differentially expressed genes were identified for lymph node metastasis and TNM stages, respectively. Of the 296 intersection genes, 112 were associated with nodal metastasis and/or staging. Only 25 of these 112 genes with gradient changes were involved in nodal metastasis, and 13 were involved in staging. Only one gene, RN7SL494P, might be involved in lung adenocarcinoma development and poor outcome. Finally, Cox regression results verified that age, pathology classification, radiotherapy and chemotherapy are all the independent prognostic factors. In particular, RN7SL494P was further verified to be an independent factor affecting lymph node metastasis and patient survival. Furthermore, we verified the RN7SL494P function using simulation data generated by mixing cell lines of the Cancer Cell Line Encyclopedia (CCLE) and obtained consistent results. Our findings suggest a potential clinical application of the RN7SL494P as a promising marker in the evaluation of patients with primary lung adenocarcinoma, not only for predicting nodal metastasis, but also for the prognosis of the outcome.

## Introduction

Lung adenocarcinoma, a histological subtype of non-small cell lung cancer (NSCLC), arises when healthy cells change and uncontrolled growth occurs in the outer region of the lung. Lung adenocarcinoma is the most common type of lung cancer and accounts for approximately 40% of all lung-derived cancers [1].

Lung adenocarcinoma tends to develop in smaller airways, such as bronchioles, and develops more slowly than any other types of lung cancer. Once cancerous tissues begin to grow, cancer cells may slough off. These cells may be carried in the blood or float in the lymph fluid that encompasses the lung tissue [2]. The lymph flows through lymphatic vessels into collecting lymph nodes [3, 4]. When a cancer cell spreads to a lymph node or passes through the bloodstream to a distant body site, it is called metastasis.

The Cancer Genome Atlas (TCGA) project was started in 2006 [5] and a joint research project between the National Human Genome Research Institute and the National Cancer Institute. In the current study, we performed a comprehensive screening of TCGA databases for transcriptome and clinical data regarding nodal metastasis and TNM staging for patients with lung adenocarcinoma. According to the primary results, we further verify the gene(s)’ function in independent data sets from the Cancer Cell Line Encyclopedia (CCLE) project [6].

## Results

### Differentially expressed genes in lung adenocarcinoma

Gene differential expression analysis between lung adenocarcinoma tissues and matched normal controls identified a total of 13,118 genes that were differentially expressed of which 2800 were down-regulated and 10,318 were up-regulated. The top 10 most significantly down-regulated and top 10 most significantly up-regulated genes are shown in Additional file 1: Table S1. We included all the significantly up-regulated and down-regulated mRNAs to generate a heatmap and volcanic map to demonstrate their relative expression levels (Additional file 2: Figure S1A, B).

### GO and KEGG analyses of differentially expressed genes

**Fig. 1:**
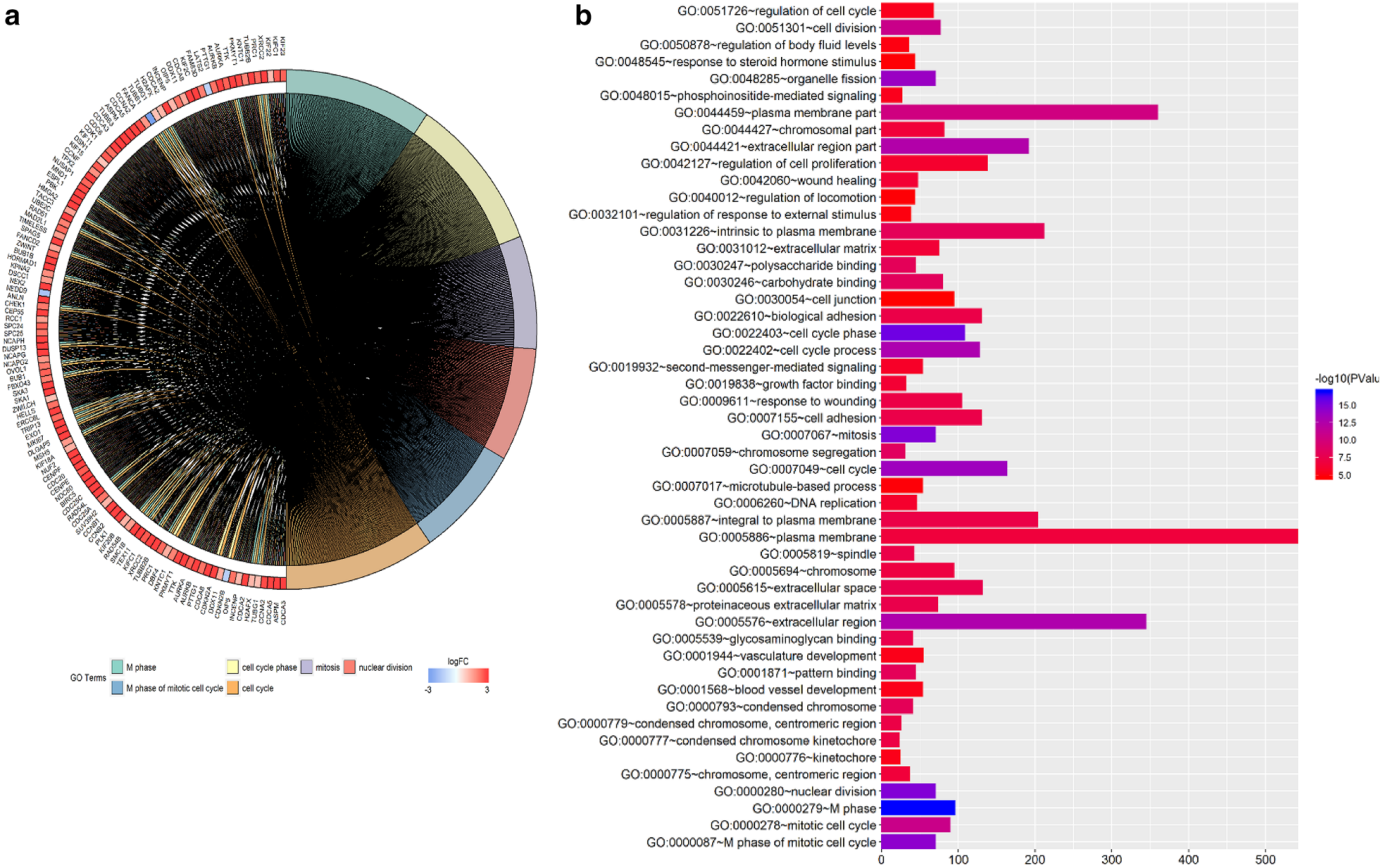
GO analyses of all differentially expressed genes in lung adenocarcinoma. **a** The biological functions, biological processes or cellular components in *DAVID* database by GOplot analysis. **b** The enrichment of differentially expressed genes

We conducted GO analysis for all the differentially expressed genes in the lung adenocarcinoma cases in the current study and found that the gene RN7SL494P was not involved in any biological functions or processes in the DAVID database, nor was it related to any cellular components of the database (Fig. 1a, b). KEGG pathway analysis and KOBAS was used to functionally annotate the differentially expressed genes. After identifying the key KEGG pathways, we determined that RN7SL494P was not associated with any of the KEGG pathways (Additional file 3: Table S2). Functional annotation of the differentially expressed genes using the clusterProfiler Supplement R package also failed to identify any RN7SL494P-related KEGG pathways (Additional file 4: Table S3). GO analysis results showed that upregulated DEGs were significantly enriched in extracellular exosome, membrane, and mitochondrion (Fig. 1a). Downregulated DEGs were mainly significantly enriched in the cytoplasm, nucleus, cytosol, nucleoplasm, and protein binding (Fig. 1b). Therefore, we concluded that a single gene functional enrichment method associated with the specific gene would be used as a subsequent step of the study.

### Differentially expressed genes associated with nodal metastasis or TNM stage

Based on the features of lymph node metastasis for the subjects listed in Additional file 5: Table S4, a total of 406 differentially expressed genes were identified. Of the differentially expressed genes, 312 were significantly up-regulated and 94 were significantly down-regulated (Additional file 2: Figure S1C, D). The top 10 most significantly down-regulated and top 10 most significantly up-regulated genes associated with cancer metastasis are shown in Table 1. Similarly, the TNM staging-related differentially expressed genes are shown in Additional file 2: Figure S1E, F with the top 10 most significantly down-regulated and top 10 most significantly up-regulated genes shown in Table 1.

**Table 1:** The top 10 significant down- and up-regulated genes associated with lymph node metastasis or TNM stages

	Genes	logFC	logCPM	p value	FDR
Lymph node metastasis					
Down-regulated	7SK	− 6.38529	5.376253	1.92E−54	1.62E−50
	SNORA73B	− 4.89863	3.941633	2.30E−47	1.29E−43
	SNORD17	− 4.59669	2.395325	2.50E−44	1.20E−40
	SCARNA6	− 4.33589	− 0.05263	1.57E−42	5.85E−39
	SCARNA5	− 6.21022	2.186735	1.80E−42	6.05E−39
	SCARNA10	− 5.72043	1.274605	4.91E−41	1.45E−37
	MSTN	− 4.5735	1.589033	3.65E−39	9.44E−36
	SCARNA7	− 3.88216	− 0.07935	7.12E−37	1.68E−33
	SCARNA13	− 3.0861	0.701175	3.84E−36	7.73E−33
	RNU4-1	− 6.06981	1.417159	3.91E−36	7.73E−33
Up-regulated	NNAT	3.773884	2.325209	2.37E−89	7.97E−85
	LRRC38	5.827189	1.230182	1.32E−68	2.23E−64
	VSX2	4.728637	− 1.57565	1.85E−55	2.07E−51
	AC087257.2	3.860068	− 2.07862	1.84E−52	1.24E−48
	LINC01433	3.163173	− 2.46671	3.82E−43	1.61E−39
	FAM205C	3.293196	− 2.91757	7.49E−37	1.68E−33
	AL161668.1	4.428092	− 3.68113	1.56E−35	2.77E−32
	RTP1	3.811513	− 2.18471	1.65E−34	2.64E−31
	GSG1L2	4.357816	− 3.36664	1.11E−31	1.44E−28
	CALB1	3.446571	3.71567	4.30E−31	5.16E−28
TNM stages					
Down-regulated	7SK	− 6.062979794	5.353737093	6.74E−31	6.13E−28
	SNORA73B	− 4.647298488	3.923197774	1.76E−27	1.26E−24
	SNORD17	− 4.325760083	2.373029662	1.51E−25	9.39E−23
	SCARNA5	− 5.981744956	2.167025263	5.95E−25	3.63E−22
	SCARNA6	− 4.052245334	− 0.070346602	2.75E−24	1.62E−21
	SCARNA10	− 5.377554867	1.251887603	1.35E−23	7.42E−21
	MSTN	− 4.340706495	1.520356478	1.70E−23	8.96E−21
	SCARNA7	− 3.7237236	− 0.10084326	2.81E−22	1.26E−19
	RNU4-1	− 5.712216563	1.396659528	6.97E−21	2.79E−18
	RNU4-2	− 5.357340472	2.502793386	1.26E−20	4.94E−18
Up-regulated	PPIAP46	4.012250624	− 0.902056299	1.92E−100	6.46E−96
	HNRNPA1P52	3.896195799	− 1.852379148	4.95E−96	8.32E−92
	LRRC38	6.291094962	1.168289657	3.92E−92	4.39E−88
	AC087257.2	4.527651209	− 2.097876396	1.53E−81	1.28E−77
	VSX2	5.232030072	− 1.594431836	1.90E−76	1.28E−72
	PSG11	7.901940389	− 1.563148972	2.93E−58	1.64E−54
	FAM205C	3.883821429	− 2.930755861	6.97E−55	3.35E−51
	FXNP2	3.718917178	− 3.212051642	1.45E−53	6.09E−50
	MARCH4	2.823672408	0.803655353	1.61E−45	6.02E−42
	RTP1	4.254620305	− 2.219012962	5.41E−45	1.82E−41

### Overlapping differentially expressed genes associated with nodal metastasis and TNM stages

**Fig. 2:**
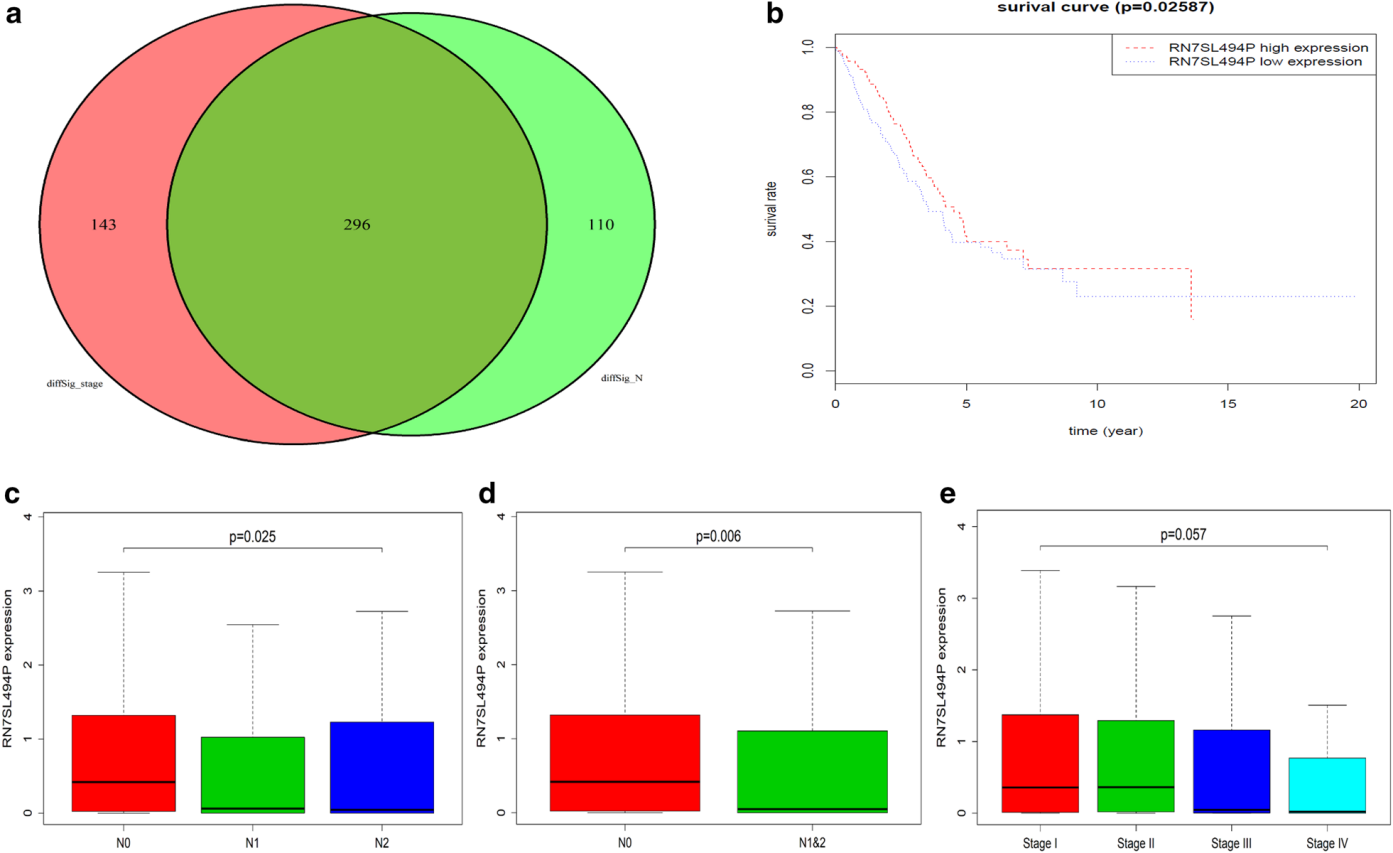
The overlapping differentially expressed genes associated with nodal metastasis and TNM staging. **a** The venn diagram of differentially expressed genes between nodal metastasis and TNM staging. **b** Survival analysis of differentially expressed RN7SL494P associated with nodal metastasis. **c** Kruskal–Wallis test for differentially expressed RN7SL494P associated with the gradient changes on lymph node metastasis (N0 vs. N1 vs. N2). **d** Kruskal–Wallis test for differentially expressed RN7SL494P associated with the gradient changes on lymph node metastasis (N0 vs. N1 and N2). **e** Kruskal–Wallis test for differentially expressed RN7SL494P associated with the gradient changes on TNM staging

Venn diagram analysis was performed to visualize the overlapping differentially expressed genes between lymph node metastasis and TNM stages. The VennDiagram R package was used and 296 overlapping genes were identified (Fig. 2a).

### Gradient changes of differentially expressed genes associated with nodal metastasis and TNM stages

We analyzed the gradient changes of differentially expressed genes in lymph node metastasis (from N0 to N2) and TNM stage (from I to IV) using the Kruskal–Wallis test. Since there were only two samples with a metastasis score of N3, this subgroup was not considered in this analysis. A total of 112 differentially expressed genes were associated with the gradient changes of lymph node metastasis, TNM stage, or metastasis and TNM stage (Table 2). Among the 112 differentially expressed genes, 25 were associated with lymph node metastasis, 13 with TNM stage, and 7 genes (SCARNA7, AC105999.2, RANBP20P, RN7SL151P, SYNPR, AL512638.1, and TMIGD1) were associated with both lymph node metastasis and TNM stage.

**Table 2:** The gradient changes of differentially expressed genes associated with lymph node metastasis or TNM stages with the Kruskal–Wallis test, and the survival analysis of patients with the differentially expressed genes

Genes	Lymph node metastasis (N0–N1–N2)		TNM stages		Log-rank test
			(I–II–III–IV)		
	Gradient change	*p*	Gradient change	*p*	*p*
NNAT	NA	0.019	NA	0.025	–
VSX2	Yes, downtrend	0.008	–	0.586	0.08025
SCARNA7	Yes, downtrend	0.011	Yes, downtrend	0.018	0.34227
AL161668.1	NA	0	NA	0.002	–
SNORA12	NA	0.013	NA	0.003	–
GSG1L2	Yes, upward	0	–	0.604	0.36278
CYP2B6	–	0.287	NA	0.032	–
ALB	–	0.197	NA	0.008	–
VN1R35P	Yes, upward	0.003	–	0.157	0.08025
SNORA71A	NA	0.04	–	0.842	–
AL451054.3	NA	0	NA	0.012	–
AC105999.2	Yes, upward	0.042	Yes, upward	0.012	0.13752
RN7SL3	Yes, upward	0.048	–	0.266	0.09487
LINC01819	Yes, downtrend	0.016	NA	0.021	–
RANBP20P	Yes, downtrend	0.019	Yes, downtrend	0.016	0.07001
RNU5A-1	–	0.066	Yes, downtrend	0.015	0.75953
RN7SKP255	–	0.101	NA	0.005	–
AL513304.1	Yes, upward	0.019	–	0.073	0.37489
HIST1H4F	–	0.191	NA	0	–
RN7SKP203	NA	0.006	–	0.334	–
HIST1H4L	–	0.342	NA	0.048	–
RN7SL769P	NA	0.01	–	0.116	–
RN7SL151P	Yes, downtrend	0.006	Yes, downtrend	0.009	0.28316
GKN1	NA	0.039	–	0.272	–
FXNP2	NA	0.006	–	0.508	–
RNY3	NA	0.003	–	0.067	–
AC112495.1	Yes, downtrend	0.012	NA	0.002	0.88522
SYNPR	Yes, downtrend	0.034	Yes, downtrend	0.002	0.14163
RN7SL480P	Yes, downtrend	0.03	–	0.169	0.97413
RN7SL116P	Yes, downtrend	0.019	–	0.057	0.71102
AC036111.1	NA	0.004	–	0.195	
RNA5-8SP2	NA	0	–	0.088	
RN7SL300P	NA	0.026	–	0.079	
HIST1H2AH	Yes, upward	0.014	NA	0.012	0.89036
PSG11	–	0.126	NA	0.002	
GLRA4	Yes, downtrend	0.003	–	0.322	0.08082
RN7SL359P	NA	0	–	0.052	–
AL135929.2	NA	0.006	–	0.14	–
CYP11B1	NA	0.029	–	0.123	–
RN7SL342P	NA	0.02	–	0.062	–
SPAG11B	Yes, upward	0.028	–	0.064	0.54783
RN7SL732P	NA	0.005	–	0.082	–
CYP1D1P	NA	0	NA	0.002	–
RN7SL791P	NA	0	NA	0.002	–
RN7SKP189	NA	0.002	–	0.696	–
RN7SKP71	Yes, downtrend	0.011	NA	0.025	0.24259
RN7SL217P	NA	0.029	NA	0.041	–
RN7SL272P	NA	0	NA	0.016	–
RHOXF2B	NA	0	–	0.093	–
RN7SL464P	NA	0.003	–	0.214	–
CRISP1	NA	0.007	–	0.074	–
FGF4	–	0.379	NA	0.019	–
CRP	NA	0.026	–	0.066	–
PSG2	–	0.347	NA	0.03	–
RN7SL197P	NA	0.017	–	0.644	–
RN7SL646P	NA	0.003	–	0.111	–
RN7SL554P	NA	0.001	–	0.317	–
PPP1R3A	NA	0.009	–	0.226	–
RN7SL597P	–	0.056	NA	0.017	–
RN7SL308P	NA	0.001	NA	0.003	–
AC106872.1	NA	0	NA	0.003	–
AL135929.1	NA	0.007	–	0.086	–
AL512638.1	Yes, upward	0.002	Yes, upward	0	0.80925
RN7SL711P	–	0.104	Yes, downtrend	0.022	0.6968
HMGB3P18	NA	0.018	NA	0.022	–
RN7SL126P	NA	0.021	–	0.106	–
RN7SL630P	NA	0.002	–	0.066	–
RN7SL494P	Yes, downtrend	0.025	–	0.057	0.02587
RN7SL7P	NA	0.024	–	0.23	–
RN7SL786P	NA	0.021	–	0.118	–
AC108515.1	NA	0	NA	0.005	–
RN7SKP185	NA	0.023	Yes, downtrend	0.02	0.66366
RN7SKP90	NA	0	Yes, downtrend	0.017	0.91288
AC008808.2	–	0.814	NA	0.024	–
RN7SL390P	NA	0.012	–	0.445	–
SCARNA3	NA	0	NA	0.007	–
MIR124-2HG	NA	0.002	NA	0.012	–
RN7SL297P	NA	0.001	NA	0.002	–
RNU1-88P	NA	0.004	–	0.35	–
RN7SL314P	NA	0.078	NA	0.038	–
RN7SL575P	NA	0.049	–	0.272	–
RN7SL302P	NA	0.04	–	0.099	–
AL513475.2	NA	0.046	–	0.401	–
KRT38	–	0.148	Yes, upward	0.031	0.30421
OR4A16	NA	0.004	NA	0.003	–
FRG2	NA	0.003	–	0.699	–
LINC02557	NA	0.001	–	0.462	–
LINC01221	NA	0.002	–	0.076	–
AC012065.1	Yes, upward	0	NA	0	0.25382
LINC01040	NA	0.014	NA	0.024	–
IGLV3-26	NA	0.003	NA	0.011	–
CRCT1	Yes, upward	0.019	NA	0.013	0.51194
GAGE12 J	NA	0.017	NA	0.007	–
CELA3A	Yes, downtrend	0.035	NA	0.003	0.60893
RN7SL260P	NA	0.005	–	0.102	–
AC245291.3	–	0.105	NA	0.018	–
AC105031.2	Yes, upward	0.001	NA	0.013	0.88735
AC245128.1	NA	0.008	NA	0.043	–
AC008517.1	NA	0.002	–	0.357	–
DRAXINP1	–	0.111	NA	0	–
RN7SL14P	NA	0.032	–	0.214	–
DDX11L16	NA	0.002	NA	0.02	–
ANHX	NA	0.043	NA	0.007	–
FAM9A	NA	0.018	NA	0	–
TMIGD1	Yes, upward	0.001	Yes, upward	0.027	0.42473
PSG7	–	0.251	Yes, upward	0.001	0.74669
AC105460.1	NA	0.01	NA	0.001	–
AC080128.1	–	0.215	NA	0.036	–
BX510359.3	–	0.064	NA	0.002	–
AL139002.1	NA	0.022	–	0.747	–
MIR3976HG	–	0.195	NA	0.002	–
SPAG11A	NA	0.003	NA	0.008	–

*d* the deleted base, *P*_corrected_ multiple testing by the Bonferroni correction, *NA* not applicable

### Survival rates and differentially expressed genes associated with nodal metastasis and TNM stage

We analyzed patient survival time relative to all 30 differentially expressed genes that were associated with the gradient changes on lymph node metastasis and/or TNM stage. Only one gene (RN7SL494P) was found to correlate with patient survival time (Table 2 and Fig. 2b). RN7SL494P was also associated with the gradient changes of lymph node metastasis with *p* = 0.02587 for N0 vs. N1 vs. N2 (Fig. 2c) and *p* = 0.006 for N0 vs. N1 vs. N2 (Fig. 2d). However, RN7SL494P was not associated with the gradient changes of TNM stage (*p* = 0.057; Fig. 2e).

### sGSEA of pathways

**Fig. 3:**
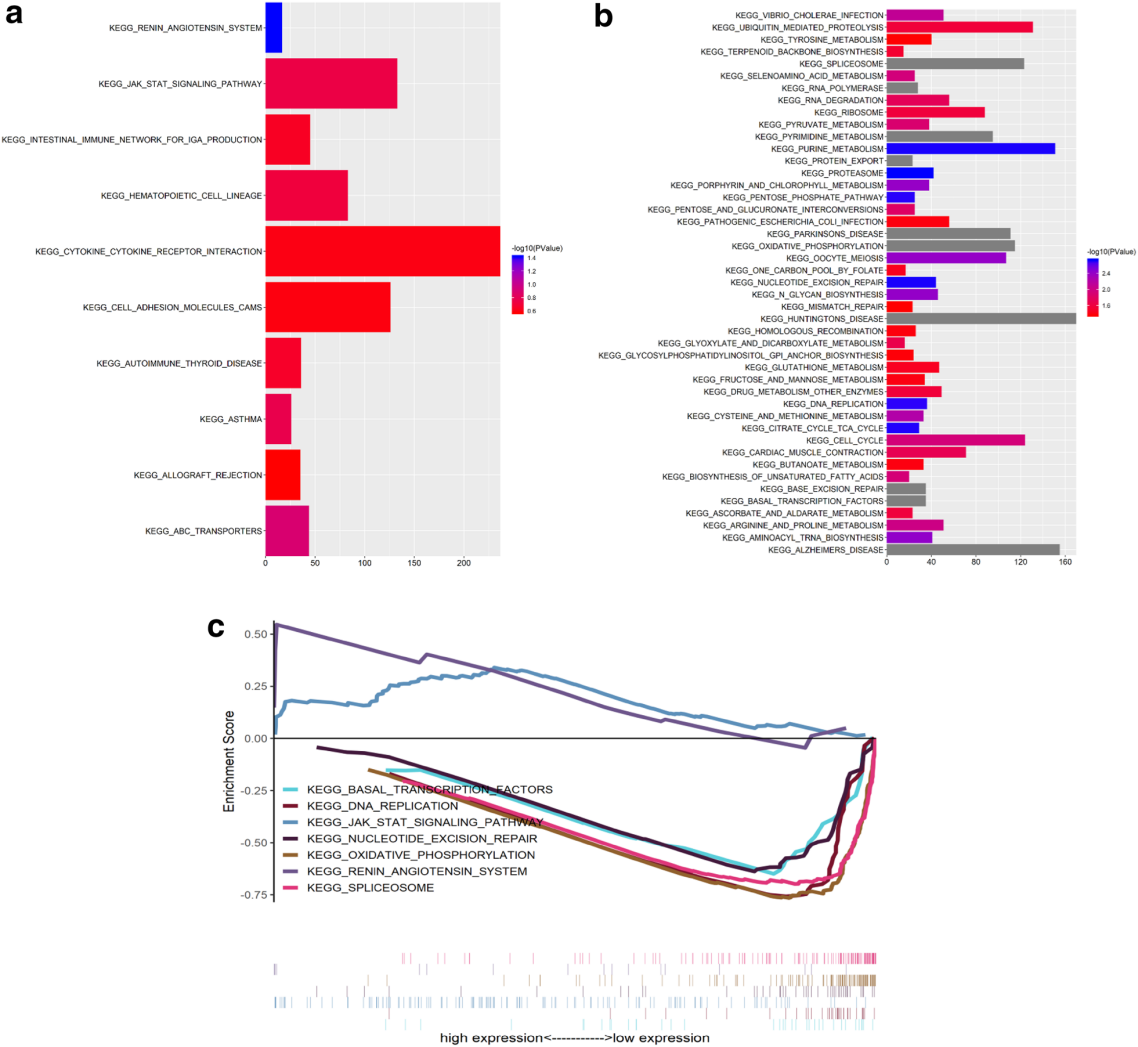
sGSEA analyses. **a** The genes co-expressed with higher expressions of RN7SL494P were enriched in biological pathways associated with KEGG_RENIN_ANGIOTENSIN_SYSTEM. **b** The genes co-expressed with lower expressions of RN7SL494P were enriched in 45 biological pathways. **c** The typical results of co-expressed with higher or lower expressions of RN7SL494P

Evaluation of the associations between RN7SL494P expression and any cancer-related pathways was performed and renin angiotensin system, JAK-STAT signaling pathway, et al. were the enriched pathways associated with higher expression of the gene RN7SL494P (Fig. 3a). On the other hand, the genes co-expressed with the low-expression of RN7SL494P were associated with biological or pathological pathways including basal transcription factors, spliceosome, oxidative phosphorylation, nucleotide excision repair, DNA replication and among others (Fig. 3b). These typical results are shown on a GSEA diagram at the same time (Fig. 3c). These findings suggested that low-expression of RN7SL494P might be associated with cancer development and poor outcome in patients with lung adenocarcinoma.

### Cox regression models

Univariate Cox analysis found that the increased expression of RN7SL494P would reduce the risk of death in patients (HR 0.78, *p* = 0.020). The patients who did receive radiotherapy, or who had a higher grade of pathology, or who had metastasis, or who had lymph node involvement, had a greater risk of death (all HR > 1, all p < 0.05) (Additional file 6: Table S5).

**Fig. 4:**
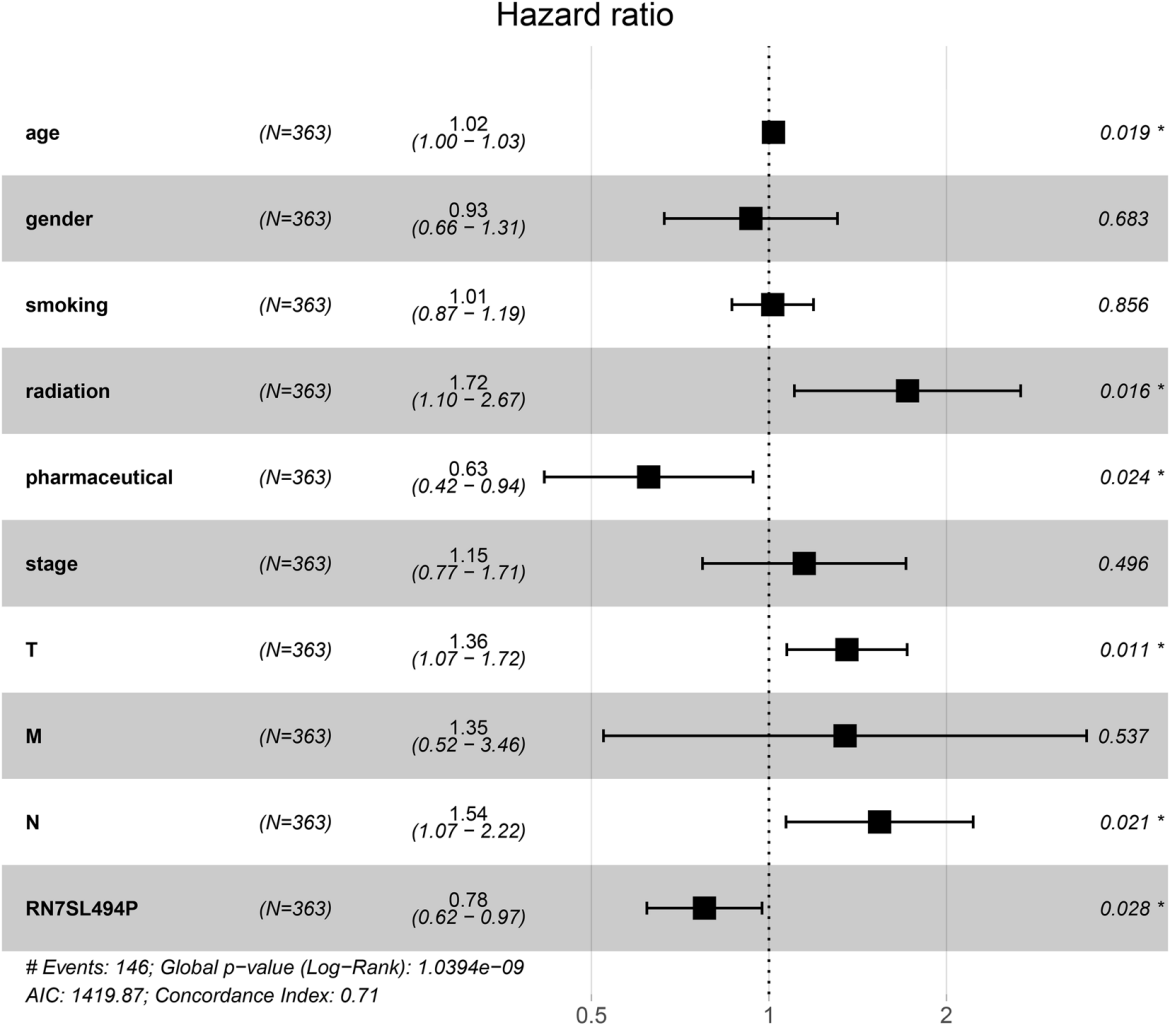
The multivariate Cox regression analysis of related clinical parameters and RN7SL494P in lung adenocarcinoma

In multivariate Cox regression analysis, we found the expression of RN7SL494P still was an independent prognostic factor (HR 0.78, p = 0.028). This further proves that this gene is a prognostic factor of lung cancer. In addition, age, stage_T and stage_N were the fisk factors, these further suggest that lymph node metastasis will lead to a worsening prognosis in patients with lung adenocarcinoma. Interestingly, the effects of radiotherapy and chemotherapy may be reversed, that is, radiotherapy may result in reduced efficacy and poor prognosis; but chemotherapy can significantly extend the survival time of such patients (Fig. 4).

### Co-expressions genes of RN7SL494P in CCLE

**Fig. 5:**
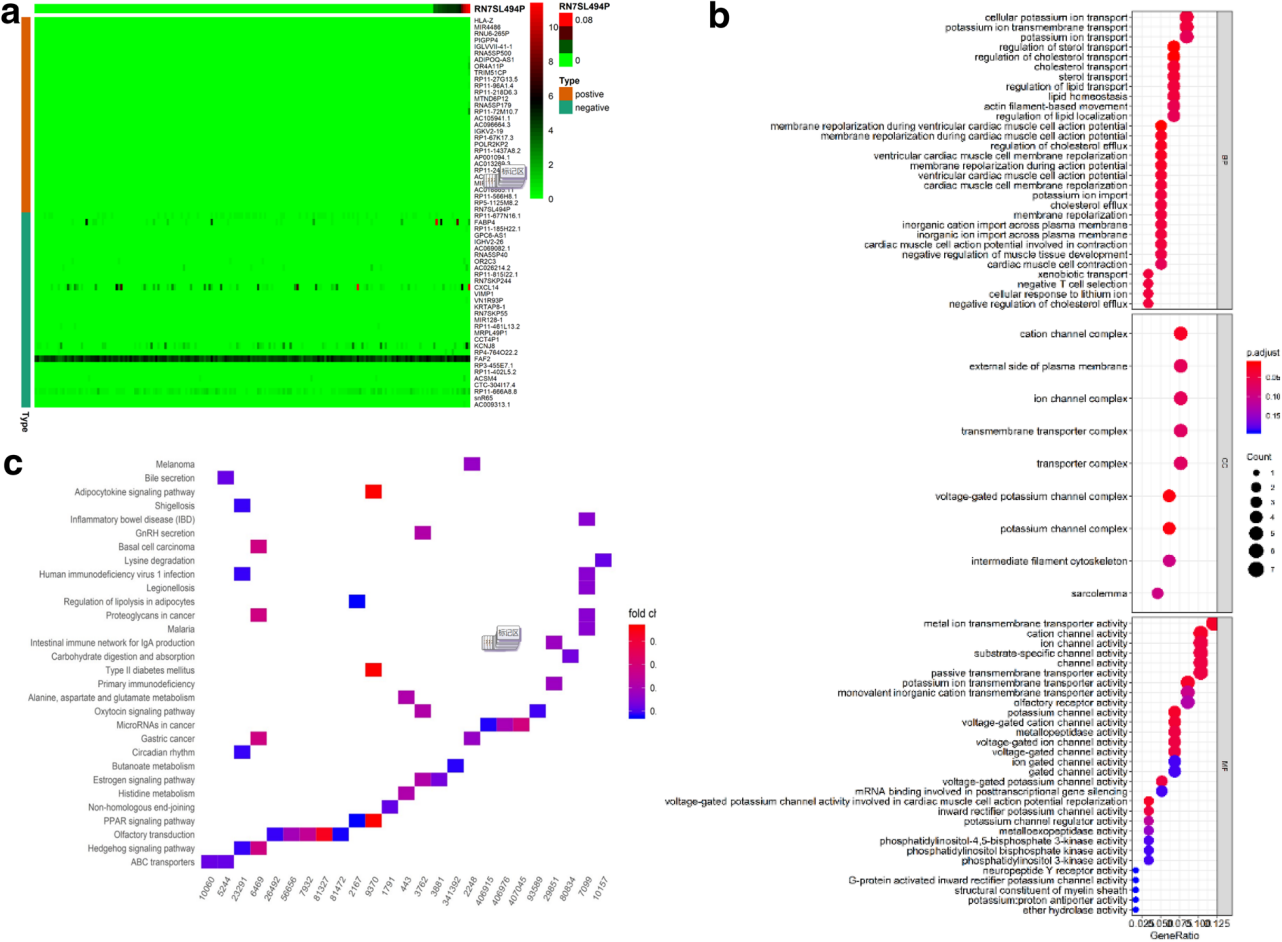
The co-expressions genes of RN7SL494P and the functional verification in the CCLE. **a** The co-expression genes of RN7SL494P. The 30 up co-expression genes and 30 down co-expression genes were selected to construct a co-expression heatmap. **b** The GO analysis of the co-expression genes of RN7SL494P. **c** KEGG analyses of the biological functions and pathways. The co-expression genes of RN7SL494P were mainly enriched in ABC transporters, Hedgehog signaling pathway, PPAR signaling pathway, and Non-homologous end-joining (p < 0.05)

We downloaded the lung cancer cell lines’ raw counts of the expression profiling from the CCLE database. The co-expression genes with RN7SL494P were calculated with a 0.2 co-expression coefficient threshold. The 30 up co-expression genes and 30 down co-expression genes were selected to construct a co-expression heatmap (Fig. 5a).

### The functional verification of enrichment and pathway of the co-expression genes in CCLE

GO analysis results showed that the above co-expression genes of RN7SL494P were significantly enriched in cholesterol and lipid transport and homeostasis, cell membrane transport function, and so on (Fig. 5b).

KEGG analyses were performed to investigate the biological functions and pathways associated with the RN7SL494P identified. The results show that the co-expression genes of RN7SL494P were mainly enriched in ABC transporters, Hedgehog signaling pathway, PPAR signaling pathway, and non-homologous end-joining (p < 0.05) (Additional file 7: Table S6, and Fig. 5c).

## Discussion

Many patients are diagnosed with cancer metastasis, which usually makes treatment more difficult. The 5-year survival rate for patients with metastatic lung cancer is approximately 1% [7]. When tumors spread outside the lungs, they may be difficult to successfully treat and cure. Since no single best treatment exists for patients with metastatic lung cancer, the choice of treatment strategies depends on the tumor location, size, and stage, as well as the cancer subtype and the lymph nodes involved.

Scientists and clinicians have attempted to exploit methods that allow cancer patients to be screened for metastasis. The main goal of screening is to reduce the number of people that die from cancer, especially metastatic cancer. To investigate the “drive genes” in metastatic lung adenocarcinoma, we examined the differentially expressed genes in the RNA-seq repository data of TCGA. We comprehensively analyzed gene expression in patients included in the database that had lung adenocarcinoma, especially gene expression in the course of tumor metastasis.

We identified the differentially expressed genes associated with lymph node metastasis and TNM stage in lung adenocarcinoma. We also found that the gene RN7SL494P not only possessed the above characteristics, but also demonstrated prognostic significance for metastatic lung adenocarcinoma. Subsequent analysis of RN7SL494P using sGSEA further demonstrated the functions and roles of RN7SL494P.

RN7SL494P (7SL) is located on chromosome 15q21.2 and belongs to a long noncoding RNA (lncRNA) class pseudogene. As a small eukaryotic cytoplasmic RNA, 7SL RNA is essential for translocation of a protein that binds to the ribosome and targets the nascent protein in the endoplasmic reticulum to be secreted or inserted into the membrane during the assembly of human signal recognition particles (SRP) [8, 9]. A study using RNA sequencing data from 11 human tissues showed that 7SL was the highest expressed non-coding RNA (ncRNAs) and was an order of magnitude higher than any mRNA detected [10]. 7SL stimulates GTPase activity of SRP and its signal receptor (SR) complex [11, 12].

Defines a set of genes based on previous biological experiments, for example, knowledge about co-expression or biochemical pathways. A recent study showed the S-structure domain of 7SL RNA is related to cellular activity in mitochondria [13]. Furthermore, in addition to the nucleotide excision repair function, the results of sGSEA demonstrated that RN7SL494P was associated with DNA replication, transcription factor, spliceosome, oxidative phosphorylation and JAK-STAT signaling pathway. Thus, RN7SL494P (7SL) may play a role in the DNA replication, transcription, translation and assembly of peptides and its dysfunction may have pathological consequences. CCLE can be a good complement to the TCGA database to improve tumor data mining. We set a validation cohort to attain external validation, and the subsequent results of RN7SL494P’s function were supportive.

We found that the high expression of RN7SL494P improved tumor survival rates in patients with lung adenocarcinoma (high-expression 41.80% vs. low-expression 39.70%; Fig. 2b). Yang et al. [14] found that the over-expression of FOXP3 is able to inhibit the transcription of 7SL mRNA by binding to its promoter and subsequently increases the translation of p53, which results in suppressing the growth of multiple tumors (lung cancer was not included). The findings from the current study suggest that the 7SL mRNA transcribed from the RN7SL494P gene may be a direct target of FOXP3 and may be enmeshed in the FOXP3/p53 feedback loop. If true, this would be consistent with the fact that there are many complex regulatory networks involved in the process of tumor formation. We speculate that the gene RN7SL494P may exhibit “inconsistent functions” in different tumor microenvironments.

In the current study, we used the information available from the TCGA database to analyze the expression of genes in patients with lung adenocarcinoma. We found that the gradient change in expression of RN7SL494P (7SL) was clearly associated with nodal metastasis. In addition, its expression correlated with its prognostic value. These findings were validated by Cox regression analysis, in particular, the function of RN7SL494P (7SL) was verified by the independent CCLE data set.

The present study presented certain limitations. Firstly, data selection from the TCGA database may potentially cause selection bias, since this is prevalent in all non-prospective, nonrandomized studies. Secondly, the CCLE database does not include clinically meaningful variables, therefore, only the function of genes and their co-expressed gene sets can be verified, but the survival time can not be verified. Thirdly, due to technical reasons, it is impossible to establish a smooth working relationship with the clinical departments of a hospital in a short term, so it is temporarily unable to conduct tests in clinical practice.

In conclusion, our results suggest that the over-expression of RN7SL494P could significantly reduce lymph node metastasis and improve the survival of patients. Meanwhile, age, pathology classifications, and treatment (radiotherapy and chemotherapy) may also affect patient survival in lung adenocarcinoma.

## Materials and methods

### The lung adenocarcinoma data and pipeline

**Fig. 6:**
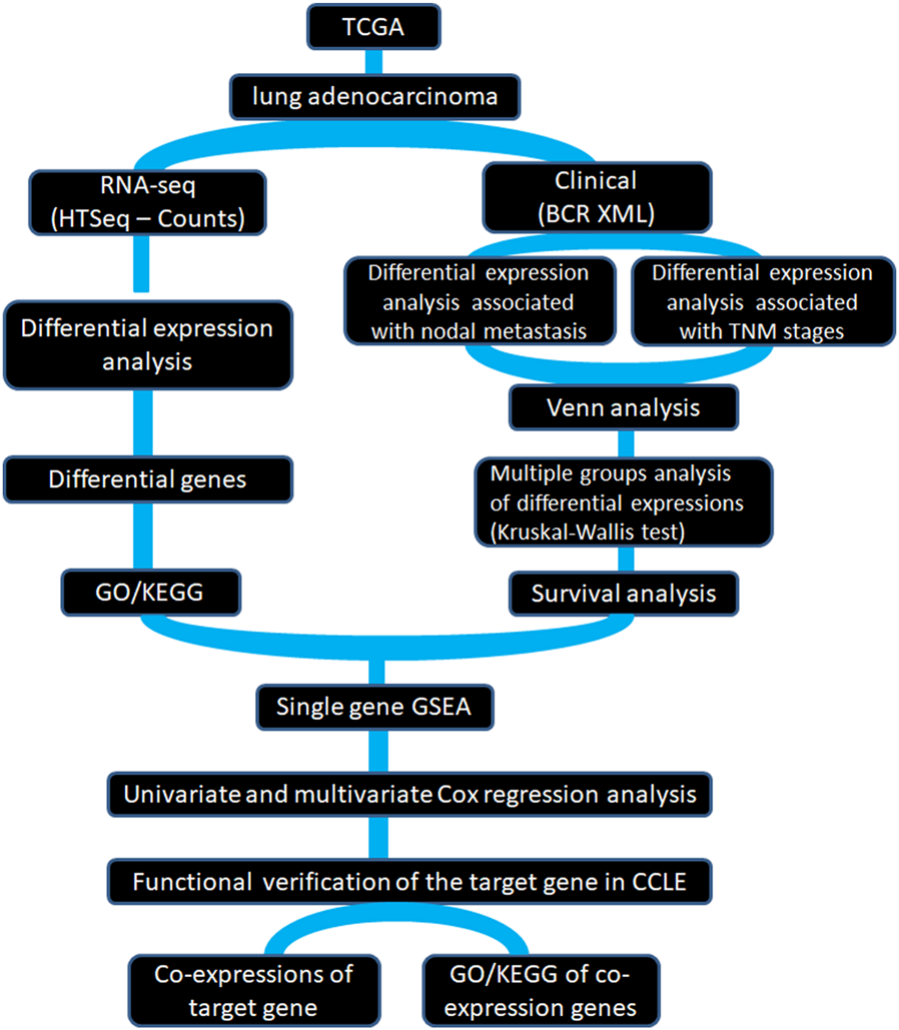
The pipeline of this study. The RNAseq data and clinical data for lung adenocarcinoma were first downloaded from the TCGA. RNA-seq data were used to analyze gene differential expression, and perform GO and KEGG functional analysis. Clinical data combined with lymph node metastasis and TNM analysis of differential genes. And the gene set associated with both lymph node metastasis and TNM stage was obtained. Then the survivals of the intersection genes were analyzed by Kruskal–Wallis algorithm to find the target gene. The function of the single gene GSEA of the target gene was then studied. The prognosis between the gene and the clinical variable was verified by the Cox regression analysis of single gene and multivariables. Finally, in the CCLE database, the co-expression genes of the target gene and the enrichment and signaling pathway analysis in which these genes are involved are further verified

The lung adenocarcinoma data (mRNA expression data and clinical data) from the National Cancer Institute’s Genomic Data Commons (GDC) portal (https://portal.gdc.cancer.gov/repository) were downloaded on August 5, 2017, using GDC-client.exe software. This provided 594 level-3 RNA-seq hits (515 cases) and 522 clinical XML datasets. The clinical data are shown in Additional file 5: Table S4. The expression data were obtained for each of the lines using Affymetrix U133 Plus 2.0 arrays from the CCLE were downloaded from the website (https://portals.broadinstitute.org/ccle) directly. The data are open to the public under certain guidelines. Therefore, confirm that all written informed consent has been achieved. The pipeline and details of the study are shown in Fig. 6.

### Differential gene expression analysis

Differential gene expression based on the RNA-seq data was analyzed using the edgeR software package [15], which involved empirical Bayesian estimations and accurate tests based on the negative binomial distributions. As edgeR suggested, genes with very low reads are often not of interest in differential expression analyses; therefore, the average count-per-million (CPM) was an important criterion used to define whether a gene was expressed at a reasonable level for inclusion. The edgeR software reported log2 fold change, log2 counts per million, the corresponding statistical significance, and their corresponding error discovery rates. The up-regulated and down-regulated differentially expressed genes were selected based on these parameters.

### Gene ontology (GO) and Kyoto Encyclopedia of Genes and Genomes (KEGG) pathway analysis

GO provides a platform for the hierarchically sorting of genes or their products by terms that fall into the three following categories: molecular functions (molecular activity), cellular component (functional gene products), and biological processes (cellular or physiological effects) [16–18]. The Database for Annotation, Visualization, and Integrated Discovery (DAVID) version 6.7 was used to perform the functional annotation analysis [19] and the ggplot2 and the GOplot R packages were used to view the results.

We used the KEGG Orthology Based Annotation System (KOBAS) algorithm [20] and the R package clusterProfiler package to analyze the KEGG pathway of gene differential expression [21]. The genes from the lung adenocarcinoma RNA-seq that exhibited significant upward and downward differential expression were analyzed. A difference with a *p*-value less than 0.05 was considered significant for the screening criterion.

### Gene Set Variation Analysis (GSVA) of KEGG pathways

A comprehensive human gene annotations document (c5.all.v5.2.symbols.gmt) for the GO function category was downloaded from the Molecular Signatures Database (MSigDB) [22]. The Gene Set Variation Analysis (GSVA) algorithm [23] was used to perform an analysis of the mRNA-SEQ data according to enrichment scores to reduce the data from an abundance of transcriptional activity at the gene level to transcriptional activity according to gene function.

### The Kruskal–Wallis test

For the analysis of differential expression associated with cancer metastasis and cancer staging, the clinical data regarding lymph node metastasis and TNM stage were selected. The Kruskal–Wallis test was used to analyze the differential expression among multiple cancer groups (N0, N1, N2, and possibly N3; and TNM stage I, II, III, and IV). As shown in Eq. 1, the Kruskal–Wallis test by grade is a nonparametric substitution method for one-way analysis of variance (ANOVA) that expands the double-sample Wilcoxon test when more than two groups are compared [24].

1 $$P = \frac{1}{{s^{2} }}\left[ {\sum\limits_{i = 1}^{k} {\frac{{R_{i} }}{{n_{i} }} - N\frac{{\left( {N + 1} \right)^{2} }}{4}} } \right]$$

where *s*^2^ is the sample variance; *k* is the number of groups; *R*_*i*_ is the total for the ith row; *n*_*i*_ is the size of the ith group; and *N* is the total number of observations.

### Survival analyses

Two risk groups were established according to the cut-off values derived from the median expression levels of the corresponding genes in the analysis of the association between gene expression and patient prognosis. The Kaplan–Meier test and the Kruskal–Wallis log-rank test were carried out to evaluate the differences in survival rates between the two risk groups. A *p*-value of less than 0.05 was considered to be statistically significant.

### Gene Set Enrichment Analysis (GSEA) and single-GSEA (sGSEA)

GSEA was used to assess the data on genomic expression levels. Relative to the median expression of the hub genes, the 515 lung cancer samples from the RNA-seq data were divided into two groups, high-expression and low-expression samples. These two GSEA groups were used to identify the potential functions of the hub genes with the c5.all.v5.2.symbols.gmt annotations being selected as the reference gene sets. Nominal differences with *p* < 0.05, false discovery rate (FDR) < 0.05, and enrichment score (ES) > 0.6 were defined as the cutoff standards.

The only gene related to the gene sets from the MSigDB [25] that was identified in the study to correlate with metastasis and prognosis (RN7SL494P) was used to determine whether the sets showed statistical differences between the low-expression and high-expression categories. The analysis was performed using the java-dependent GSEA 3.0 software package [26].

### Univariate and multivariate Cox analysis

Cox proportional risk regression analysis is applicable to quantitative prediction variables and classification variables. The aim of the model is to assess the impact of several factors on survival simultaneously. In other words, it allows us to examine how specific factors affect the incidence of specific events (e.g., infection, death) that occur at specific points in time. This rate is often called the risk rate. Predictors (or factors) are commonly referred to in the survival analysis literature as covariates. Possible variables affecting survival time and survival status of lung adenocarcinoma, including age, gender, smoking, whether to receive radiotherapy, whether to receive chemotherapy, and tumor grading, were included in univariate and multivariate Cox regression analysis to determine whether the target genes found above also affect the survival of lung adenocarcinoma.

### The functional verification of RN7SL494P in CCLE lung cancer lines

Cell line name annotation and RNA-seq data were downloaded from the CCLE database, and the “lung cancer” matrix was extracted by Perl and R. The co-expression gene set of RN7SL494P was analyzed and the co-expression heatmap was drawn. Finally, GO and KEGG functional enrichment analyses were performed on the co-expression genes of RN7SL494P.

## Supplementary information

**Additional file 1: Table S1.** The top 10 significant down- and up-regulated genes associated with lung adenocarcinoma.

**Additional file 2: Figure S1.** The differentially-expressed genes analyses. A, B Total differential expression genes in lung adenocarcinoma (A heatmap, B volcano map); C, D The differential expression genes in nodal metastasis (C heatmap, D volcano map); E, F The differential expression genes in TNM staging (E heatmap, F volcano map).

**Additional file 3: Table S2.** The KEGG pathway differential gene function annotation with Kobas algorithm.

**Additional file 4: Table S3.** The KEGG analysis of differentially expressed-genes with clusterProfiler R package.

**Additional file 5: Table S4.** Clinical and laboratory features of the subjects included in the study.

**Additional file 6: Table S5.** The univariate Cox analysis of related clinical parameters and RN7SL494P in lung adenocarcinoma.

**Additional file 7: Table S6.** KEGG analyses of the biological functions and pathways associated with the RN7SL494P identified.

## Data Availability

The datasets used and/or analyzed during the current study are available from the first author (xiao.zhu@uga.edu) on reasonable request.
