# Supplementary material for: Transcriptome analysis reveals an important candidate gene involved in both nodal metastasis and prognosis in lung adenocarcinoma
Source: Cell Biosci. 2019 Nov 19;9:92. doi: 10.1186/s13578-019-0356-1 (PMC6862851; doi:10.1186/s13578-019-0356-1)
Supplement: Supplementary file 1 — Additional file 1: Table S1. The top 10 significant down- and up-regulated genes associated with lung adenocarcinoma. [file 13578_2019_356_MOESM1_ESM.doc]

Table S1. The top 10 significant down- and up-regulated genes associated with lung adenocarcinoma.

|  | **Genes** | **logFC** | **logCPM** | **P value** | **FDR** |
| --- | --- | --- | --- | --- | --- |
| Down-regulated  Up-regulated | RTKN2  FAM107A  OTUD1  EPAS1  TEK  S1PR1  RGCC  SEMA3G  SPAAR  STX11  PYCR1  TEDC2  IQGAP3  ETV4  FAM83A  TOP2A  GOLM1  SAPCD2  TMEM184A  ALDH18A1 | -4.068647319  -4.529447985  -2.103762476  -2.695426362  -3.244335981  -2.841234568  -2.871276087  -3.203024311  -2.710967738  -2.992538831  3.72498895  3.528502981  3.632830071  3.853849662  6.825890252  3.886140596  2.662542406  3.78667196  3.148020595  1.633757577 | 5.46758936  5.196616095  4.564398143  9.192377662  4.34234913  5.220910855  6.177191804  3.936371095  0.421822555  3.548342519  6.81287369  2.55920153  4.869445161  5.517196991  7.149252358  6.847342295  8.154528815  3.975269302  5.406608823  6.730939458 | 4.80E-226  2.82E-213  1.27E-208  9.16E-203  5.41E-199  1.01E-197  2.42E-197  8.92E-197  4.05E-195  6.58E-194  2.96E-94  2.23E-75  8.84E-70  8.15E-69  1.27E-68  3.26E-66  5.03E-66  9.14E-66  7.89E-65  1.98E-64 | 1.68E-221  4.94E-209  1.47E-204  8.00E-199  3.78E-195  5.90E-194  1.21E-193  3.90E-193  1.57E-191  2.30E-190  6.06E-92  2.87E-73  9.63E-68  8.66E-67  1.34E-66  3.14E-64  4.83E-64  8.73E-64  7.24E-63  1.80E-62 |
