# Supplementary figures and images for: Transcriptome analysis reveals an important candidate gene involved in both nodal metastasis and prognosis in lung adenocarcinoma
Source: Cell Biosci. 2019 Nov 19;9:92. doi: 10.1186/s13578-019-0356-1 (PMC6862851; doi:10.1186/s13578-019-0356-1)

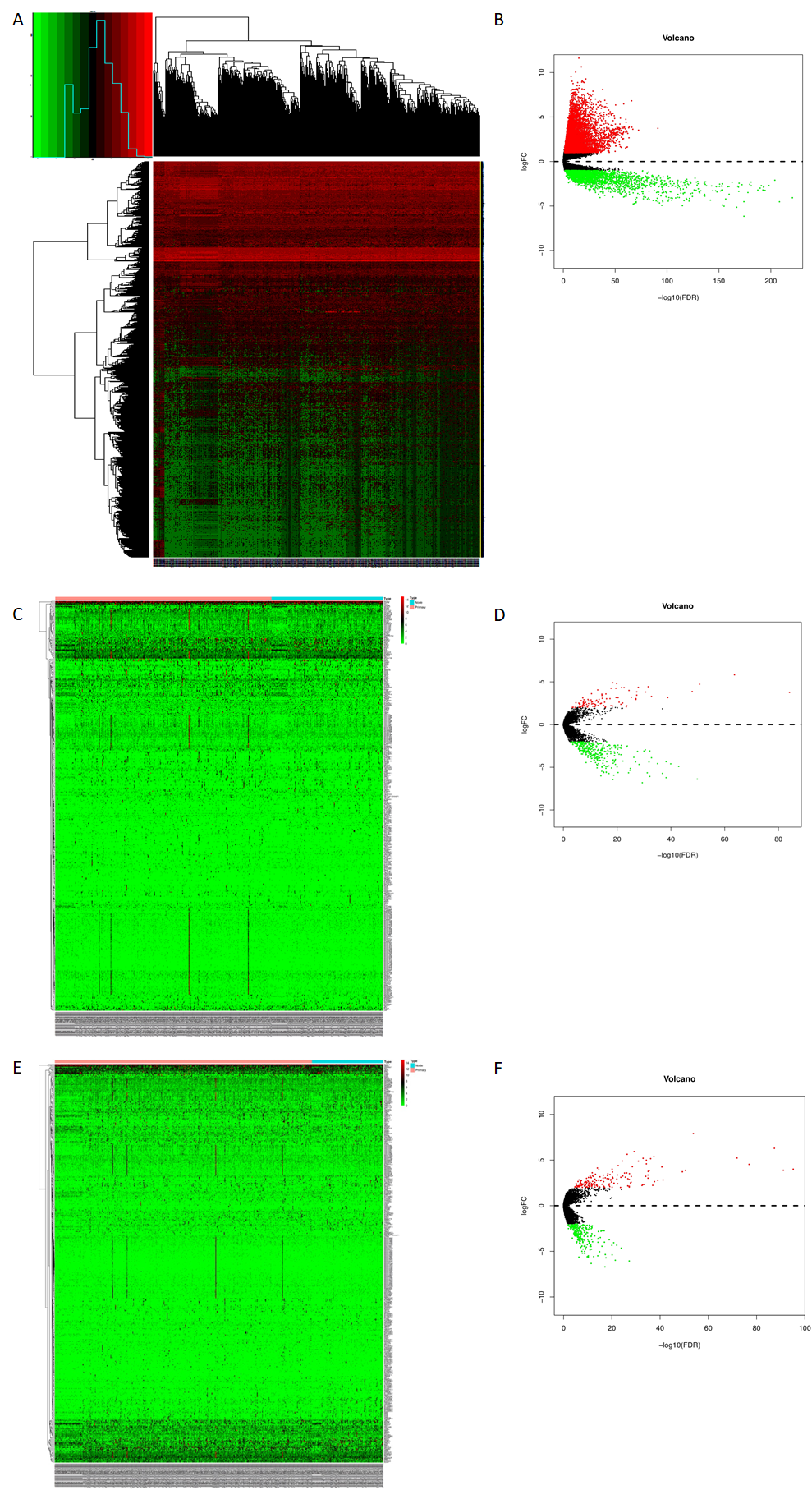

Supplement: Supplementary file 2 — Additional file 2: Figure S1. The differentially-expressed genes analyses. A, B Total differential expression genes in lung adenocarcinoma (A heatmap, B volcano map); C, D The differential expression genes in nodal metastasis (C heatmap, D volcano map); E, F The differential expression genes in TNM staging (E heatmap, F volcano map). [file 13578_2019_356_MOESM2_ESM.tif]
