# Supplementary material for: Transcriptome analysis reveals an important candidate gene involved in both nodal metastasis and prognosis in lung adenocarcinoma
Source: Cell Biosci. 2019 Nov 19;9:92. doi: 10.1186/s13578-019-0356-1 (PMC6862851; doi:10.1186/s13578-019-0356-1)
Supplement: Supplementary file 5 — Additional file 5: Table S4. Clinical and laboratory features of the subjects included in the study. [file 13578_2019_356_MOESM5_ESM.docx]

Table S4. Clinical and laboratory features of the subjects included in the study.

| Characteristics | Alive  (n=355) | Dead with tumor (n=125) | Dead tumor free (n=42) | Total (n=522) |
| --- | --- | --- | --- | --- |
| Age (ys)  Mean (SD)  Median [MIN, MAX]  Gender  FEMALE  MALE  metastasis  N0  N1  N2  N3  unknown  TNM stage  I  IA  IB  II  IIA  IIB  IIIA  IIIB  IV  unknown | 65.1 (9.8)  66 [33,88]  193 (54.37%)  162 (45.63%)  258 (72.68%)  51 (14.37%)  36 (10.14%)  2 (0.56%)  8 (2.25%)  4 (1.13%)  108 (30.42%)  109 (30.70%)  0 (0.00%)  32 (9.01%)  45 (12.68%)  36 (10.14%)  4 (1.13%)  11 (3.10%)  6 (1.69%) | 64 (10.8)  66.5 [40,84]  71 (56.80%)  54 (43.20%)  54 (43.20%)  33 (26.40%)  34 (27.20%)  0 (0.00%)  4 (3.20%)  0 (0.00%)  22 (17.60%)  19 (15.20%)  0 (0.00%)  15 (12.00%)  25 (20.00%)  25 (20.00%)  4 (3.20%)  13 (10.40%)  2 (1.60%) | 69.8 (9.8)  72 [53,85]  16 (38.10%)  26 (61.90%)  23 (54.76%)  14 (33.33%)  5 (11.90%)  0 (0.00%)  0 (0.00%)  1 (2.38%)  4 (9.52%)  12 (28.57%)  1 (2.38%)  3 (7.14%)  3 (7.14%)  13 (30.95%)  3 (7.14%)  2 (4.76%)  0 (0.00%) | 65 (10.3)  66 [33,88]  280 (53.64%)  242 (46.36%)  335 (64.18%)  98 (19.77%)  75 (14.37%)  2 (0.38%)  12 (2.30%)  5 (0.96%)  134 (25.67%)  140 (26.82%)  1 (0.19%)  50 (9.58%)  73 (13.98%)  74 (14.18%)  11 (2.11%)  26 (4.98%)  8 (1.53%) |

TNM, [tumor, nodes, metastasis-classification](http://abbr.dict.cn/tnm_2Dclassification+tumor_2Cnodes_2Cmetastasis_2Dclassification/tnm).

Clinical XML: cases=522 files=522

RNA-seq: cases=515 files=594

7 cases no RNA-seq data.
